# Impact of the three-fold channel substitution D131N on kinetics of translocation of Fe^2+^ across the protein coat is more severe for human cytosolic H-chain ferritin than for human mitochondrial ferritin

**DOI:** 10.1039/d5dt02739j

**Published:** 2026-04-01

**Authors:** Zinnia Bugg, Charlie Hazlewood, Andrew M. Hemmings, Justin M. Bradley, Nick E. Le Brun

**Affiliations:** a Centre for Molecular and Structural Biochemistry, School of Chemistry, Pharmacy and Pharmacology, University of East Anglia Norwich NR4 7TJ UK N.Le-Brun@uea.ac.uk Zinnia.Bugg@bio.chemie.uni-freiburg.de; b Centre for Molecular and Structural Biochemistry, School of Biological Sciences, University of East Anglia Norwich NR4 7TJ UK; c International Research Centre for Food and Health, College of Food Science and Technology, Shanghai Ocean University Nanhui New City Shanghai 201306 China

## Abstract

Ferritins play a key role in iron management in organisms from all kingdoms of life. Excess iron is sequestered in mineral form within the hollow protein shell and can be liberated when supply becomes restricted. The protein consists of 24 isostructural monomeric units that pack with 4-, 3-, and 2-fold symmetry. Channels through the protein coat at the 3-fold axes of ferritins localised in the cytosol of animal cells contain a strictly conserved LCDFXEX ‘twin carboxylate’ motif, and have been shown to be the major iron entry route to animal ferritins, facilitating access to the H-chain intra-subunit catalytic ferroxidase centre. In the ferritin localised to the mitochondria of animals, there is natural variation within the residues lining this channel, such that the Asp residue of the twin carboxylate motif (Asp131) is not strictly conserved. Here we report X-ray crystallographic and solution kinetic studies of the properties of D131N variants of H-chain and mitochondrial ferritins. X-ray structures revealed significant perturbation of metal binding at the three-fold channels and ferroxidase centres of H-chain ferritin, but a relatively minor effect on mitochondrial ferritin. Likewise, kinetic data showed that rapid Fe^2+^ uptake was abolished in the D131N variant of H-chain ferritin, but less severely impacted in the equivalent variant of mitochondrial ferritin. Differences were also observed in rates of mineralisation and extent of iron release in the D131N variants of the two ferritins. The implications for the physiological role of mitochondrial *versus* cytosolic ferritin are discussed.

## Abstract 2

The distinct effects on Fe^2+^ uptake of a naturally occurring Asp to Asn substitution in the three-fold channel dicarboxylate motif support different physiological roles for human mitochondrial and cytosolic ferritins.

## Graphical abstract

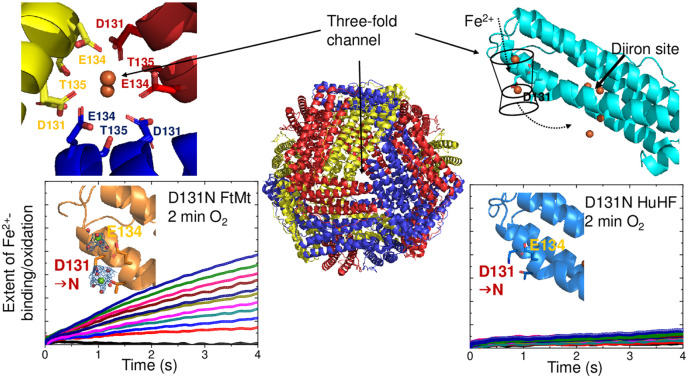

## Introduction

Iron is essential for almost all of life, being required for a broad range of fundamental processes such as respiration, DNA metabolism, amino acid synthesis and photosynthesis.^1^ These exploit the capacity of iron to undergo facile interconversion between the Fe^2+^ and Fe^3+^ oxidation states, to act as a Lewis acid catalyst and, more generally, to form stable complexes with a range of protein and non-protein ligands.^2^ The reactivity of iron, particularly toward O_2_ and its reduced forms, leading to catalysis of Haber–Weiss chemistry also means that it is potentially toxic. Therefore, to minimize the potential for deleterious activity, organisms must control the speciation of intracellular iron. The insolubility of iron at neutral pH in O_2_-containing environments^3^ makes its acquisition by biological systems challenging, meaning that catalysis of damaging reactions by iron is seldom suppressed *via* simple excretion of excess metal from cells.^4^

**Fig. 1:**
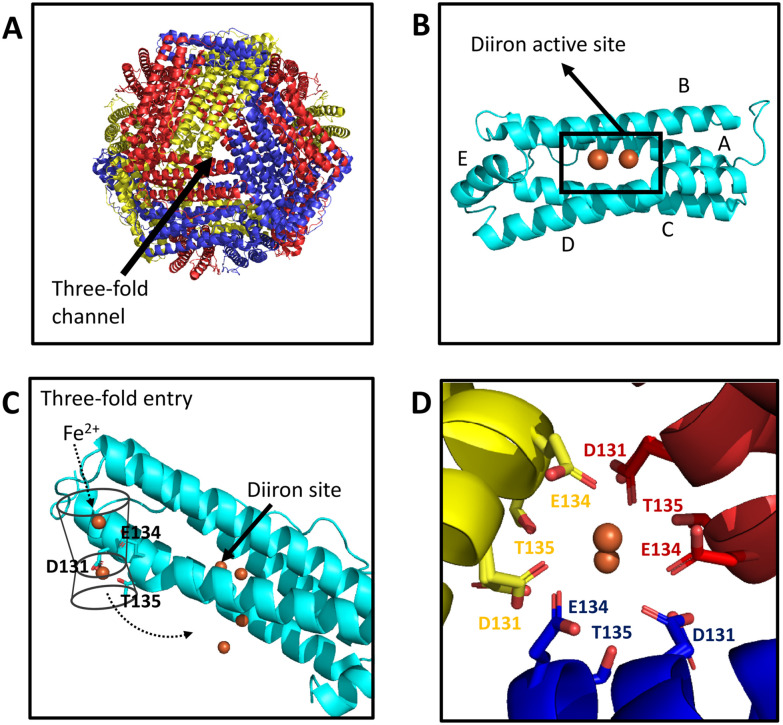
Iron entry and binding to HuHF. (A) The packing of ferritin subunits creates vertices with three-fold symmetry with channels penetrating the protein coat that form the iron entry route in HuHF. (B) Each subunit is formed from 5 helices (A–E) with the catalytic centre of H-chains buried at the centre of helices A–D. (C) A series of negatively charged residues guide Fe^2+^ from bulk solution, *via* the three-fold channel, to the diiron FOC. (D) Metal ions coordinated by Glu134 and by a combination of Asp131 and Thr135 are observed in structures of HuHF derived from X-ray diffraction data. Images produced using coordinates from PDB entry 4YKH with iron represented by orange spheres.

Ferritins are integral to the safe utilization of iron for biological processes in most organisms.^5–8^ These cage-forming proteins detoxify excess intracellular iron by storing it in the form of a ferrihydrite-like mineral within their hollow central cavity.^9^ Most ferritins consist of 24 isostructural subunits arranged as a rhombic dodecahedron (Fig. 1A). This imposes 4,3,2 symmetry upon the biological assembly, with channels at the 4-, 3- and, in prokaryotic examples, 2-fold symmetry axes. These channels, approximately 2 Å in diameter at their narrowest point,^10^ are thought to transport the metal ions and protons that are essential to ferritin activity across the 15 Å thickness of the protein coat.

Cytosolic animal ferritins consist of two different subunit types: H-chain (heavy) and L-chain (light).^9^ These subunits are isostructural, containing a four α-helical bundle (helices A–D) with a fifth, short helix (helix E) at the C-terminus (Fig. 1B), such that they can co-assemble in any ratio without affecting the topology of the resulting protein cage. The key distinction between them is that the H-chain contains the ferroxidase centre (FOC), an intra-subunit diiron site that couples the catalytic oxidation of 2 equivalents of Fe^2+^ to the reduction of O_2_ to peroxide. The L-chain lacks this site, but instead contains on its inner surface the site of iron core nucleation. In contrast, ferritins found in animal mitochondria are 24-meric homopolymers, consisting of only H-chain-type subunits that are closely related in sequence to cytosolic H-chains (for example, the mature peptide of human mitochondrial ferritin (FtMt) is 80% identical to human cytosolic H-chain ferritin (HuHF)).^11^

In cytosolic animal ferritins, Fe^2+^ enters the protein *via* the three-fold channels, which are lined with negatively charged residues and have a wide funnel-like opening which guides the metal ions to the inner surface and on towards the ferroxidase centre, Fig. 1C.^12^ In contrast, the 4-fold channels are lined predominantly by hydrophobic residues and, in general, are not involved in iron uptake. However, engineering of these channels to increase the number of negatively charged residues conferred the ability to take up Fe^2+^.^13^ The 3-fold channels are also able to facilitate iron release following reduction of the ferric mineral core,^14^ through a process by which the protein undergoes localised unfolding around the channels to promote the release of ferrous iron.^14,15^ Iron release can also occur through lysosome-mediated turnover of the protein^16^ and the physiological balance between these mechanisms is not yet understood.

High-resolution crystal structures of iron-loaded HuHF revealed hexa-aqua coordinated Fe^2+^ bound at the 3-fold channel at two distinct sites. Three symmetry-equivalent Glu134 residues coordinate a single ion (site 6), whilst a further Fe^2+^ ion is coordinated by each of the Asp131/Thr135 motifs (site 5) Fig. 1D.^12^ These residues are strictly conserved in H-chain sequences (Fig. S1) and disruption of either site by site-directed mutagenesis resulted in inhibition of Fe^2+^ oxidation, consistent with impacted iron entry.^17^

Residues Asp131, Glu134 and Thr135 of HuHF are conserved in human and most other predicted FtMts (Fig. S1). Crystal structures of iron-loaded FtMt have revealed Fe^2+^ bound at sites analogous to those of H-chain ferritins.^18^ This implies that there is likely a common mechanism, dependent on Asp131, Glu134 and Thr135, for iron entry and possibly exit *via* the three-fold channels. However, inspection of the peptide sequences of predicted FtMts, Fig. S1B, revealed that the protein from Philippine tarsier (*Carlito syrichta*), a small primate, contains Asn at the equivalent position of Asp131 in HuHF and human FtMt. This substitution of a negatively charged residue for a neutral one, without any nearby compensatory substitutions, suggests that either the tarsier FtMt three-fold channel functions without one of the key three-fold channel residues and, therefore, iron-binding sites or that rapid transport of Fe^2+^ to the FOC is not essential for FtMt function. Here, through X-ray crystallography and solution kinetic studies, we explore the effect of the three-fold channel D131N substitution on the activities of HuHF and FtMt, the best characterised examples of cytosolic H-chain and mitochondrial ferritins, respectively.

## Results and discussion

### The D131N substitution affects metal binding to HuHF to a greater extent than FtMt in structures derived from Fe^2+^/Mg^2+^ co-crystals

Formation of diffraction-quality crystals of animal ferritins requires a pH of approximately 9, limiting the solubility of Fe^2+^ due to the formation of Fe(OH)_2_.^19^ Furthermore, the 2 M concentration of Mg^2+^ ions required as precipitant acts as a competitive inhibitor of Fe^2+^ binding to the protein.^20^ Consequently, traditional methods of producing iron-enriched structures by soaking of crystals in Fe^2+^-containing well solutions failed to produce high-resolution structures with iron bound to ferritin. Two complimentary methods to overcome this difficulty have been reported. Addition of solid ferrous ammonium sulfate to drops containing crystals of HuHF or FtMt for defined time periods prior to freezing of the crystals revealed sequential occupation of a series of iron-binding sites, interpreted as transient binding sites for shuttling Fe^2+^ from bulk solution to the FOC.^12,18^ Alternatively, anaerobic crystallisation of ferritin in the presence of sufficient Fe^2+^ to out-compete Mg^2+^ leads to the formation of crystals pre-loaded with Fe^2+^ in which reactivity can be initiated by exposure to O_2_.^21^ Fe^2+^-enriched crystals of FtMt and HuHF formed *via* the latter method diffracted to ≥2.0 Å resolution and revealed similar patterns of metal ion binding to those recently reported.

**Fig. 2:**
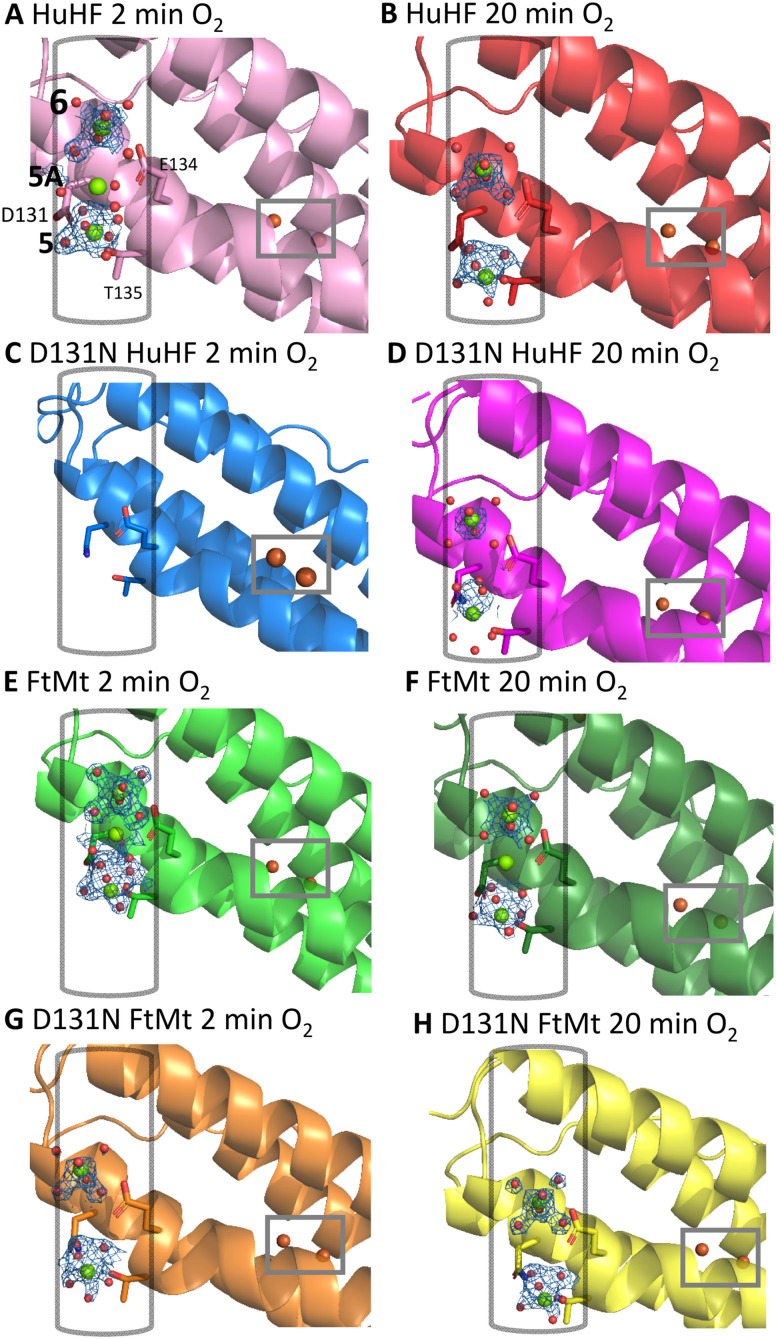
Magnesium binding to the three-fold channel of human ferritin. Magnesium ions identified within the three-fold channel from X-ray diffraction data collected from crystals of wild-type HuHF (A) and (B), D131N HuHF (C) and (D), wild-type FtMt (E) and (F) and D131N FtMt (G) and (H). Crystals were grown anaerobically in the presence of Fe^2+^ and exposed to O_2_ for 2 min (A), (C), (E) and (G) or 20 min (B), (D), (F) and (H). The FOCs in the various structures are highlighted with grey rectangles and the three-fold channels with grey cylinders. Mg^2+^ is represented as green spheres, iron as orange. Blue mesh depicts the 2*mF*_o_–*F*_c_ map contoured at 2.0*σ* up to 1.6 Å from each Mg^2+^.

**Fig. 3:**
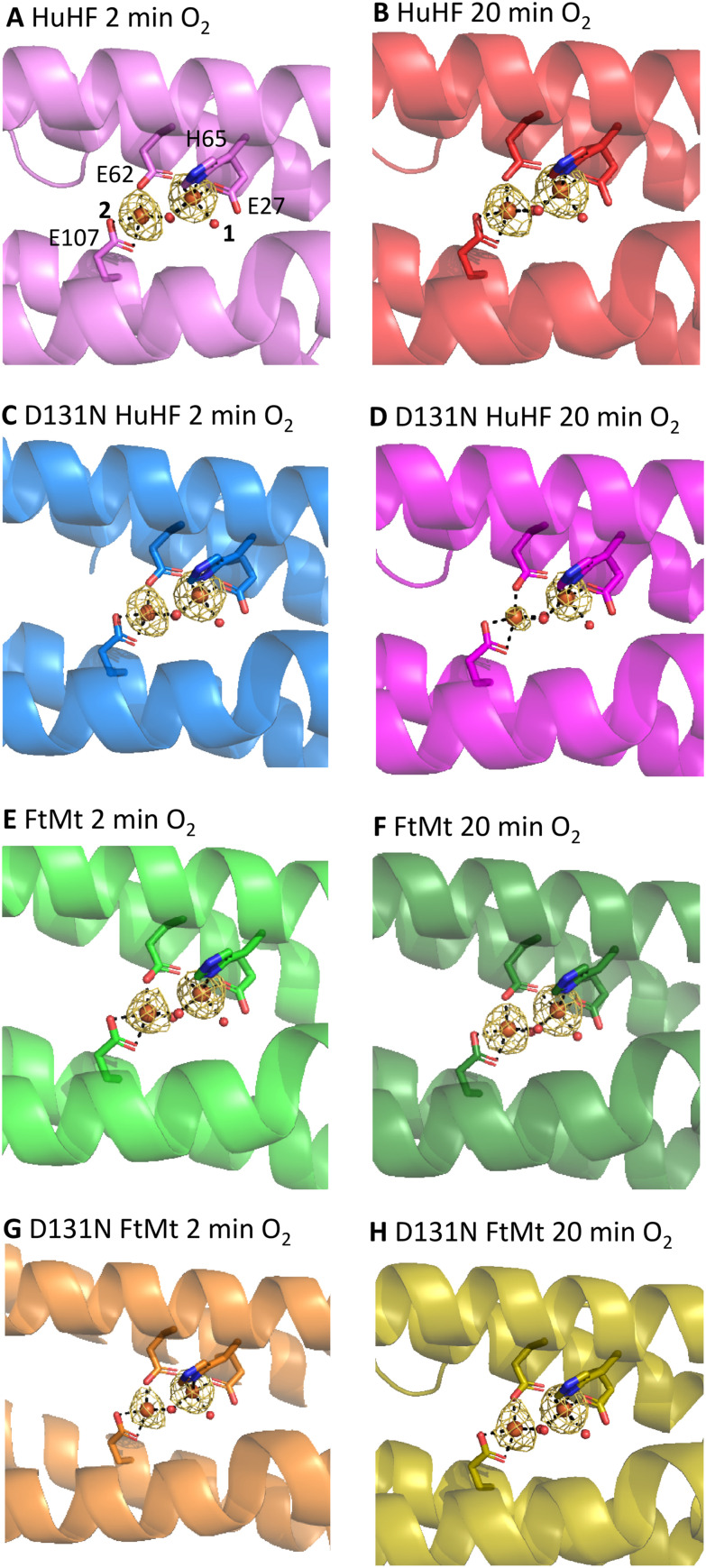
Iron binding to the FOC of human ferritins. Iron bound in the FOC of wild type HuHF (A) and (B), HuHF variant D131N (C) and (D), wild type FtMt (E) and (F) and variant D131N FtMt (G) and (H). (A), (C), (E) and (G) are structures derived from crystals exposed to O_2_ for 2 minutes, (B), (D), (F) and (H) from crystals exposed to O_2_ for 20 minutes. The sidechains of residues coordinating iron at sites 1 and 2 are shown as sticks with the residue and site numbers labelled in (A). Yellow mesh depicts the anomalous difference map contoured at 2*σ* calculated from data collected at the Fe K_α_ absorption edge.

No iron was identified with anomalous scattering at sites 5 and 6 (which are associated with Asp131 and Glu134, respectively). However, hexa-aqua coordinated Mg^2+^ can be modelled at these sites (Fig. 2), indicating that, at the concentrations used in the drops, Mg^2+^ outcompetes Fe^2+^ for binding here. Similar analysis of site 3, in close proximity to the FOC, also suggested binding of Mg^2+^ in preference to Fe^2+^ at the concentrations employed. However, binding of Fe^2+^ was apparent at the FOC (Fig. 3), at site 4 on the inner surface of the protein beneath the FOC, and at the inner surface of the 4-fold channel. Comparison of the metal ion occupancies between short and long soaking times (Table 1) revealed only minor changes, indicating that transit of substrate through the protein was sufficient to maintain the equilibrium between vacant and occupied FOC sites on extended exposure to O_2_. However, in the case of HuHF, the Bijvoet-difference Fourier map contained no peak at site 4 following extended exposure to O_2_, suggesting that iron originally bound to this site was replaced by Mg^2+^.

**Table 1:** Metal ion occupancy in HuHF and FtMt following exposure to O_2_. Fractional occupancy of ferritin metal ion binding sites defined in Fig. S2 following exposure of crystals to O_2_ for the indicated time period. An asterisk indicates that the site is occupied by Mg^2+^ rather than iron. Occupancy of the four-fold channel site and the third three-fold channel site (5A) are not listed

Soak	Site 1	Site 2	Site 3	Site 4	Site 5	Site 6
	FOC		Near-FOC		3-Fold channel	
HuHF 2 min	0.9	0.6	0.3*	0.3	0.1*	1.0*
HuHF 20 min	1.0	0.7	0.7*	1.0*	0.1*	1.0*
D131N HuHF 2 min	0.9	0.5	0.8*	—	—	—
D131N HuHF 20 min	0.6	0.2	0.6*	—	0.1*	0.6*
FtMt 2 min	1.0	0.5	0.6*	0.2	0.1*	1.0*
FtMt 20 min	1.0	0.7	0.6*	0.4	0.2*	1.0*
D131N FtMt 2 min	1.0	0.3	0.8*	0.4	0.1*	0.3*
D131N FtMt 20 min	1.0	0.5	0.7*	0.4	0.1*	0.3*

Crystals of variant D131N of both HuHF and FtMt also diffracted to ≥2.0 Å resolution. Comparison with the structures of the respective wild-type proteins revealed significant differences in the consequences of this substitution between HuHF and FtMt (Table 1). Following 2 min exposure to O_2_, sites 5 and 6 within the three-fold channel of HuHF variant D131N were vacant (as was site 4 on the inner surface of the protein). In contrast, all of these sites were occupied in FtMt variant D131N (Fig. 2). Curiously, the occupancy of site 5, coordinated by Asp131 in the wild-type protein, was unaffected whilst the occupancy of site 6 was significantly reduced in the D131N FtMt variant (Table 1). Fe^2+^ was bound to site 4 of D131N FtMt at higher occupancy than in the wild-type protein. Within the FOC, occupancy of site 2 was slightly decreased and that of site 1 unaffected by the D131N substitution in both proteins (Fig. 3).

Extending the O_2_ exposure time to 20 min had little effect on the occupancy of the metal-binding sites of D131N FtMt. In contrast, extending the O_2_ soaking time for crystals of D131N HuHF resulted in Mg^2+^ binding at sites 5 and 6. Again, the occupancy of site 5 was unaffected compared to the wild-type protein whilst that of site 6 was significantly reduced. Site 4 remained vacant in the HuHF variant protein whilst the occupancy of both FOC sites was reduced in comparison both to short O_2_ exposure of the variant and equivalent soaks of the wild-type protein.

Analysis of the B-factors of the residues in the LC(D/N)FLET motif lining the 3-fold channel (Fig. S3) revealed that for each position within the peptide sequence the thermal displacement parameters lie within 3*σ* of one another. This suggests a similar degree of static disorder at these positions across all proteins studied and therefore little effect on the rotomer populations sampled at room temperature, though it is recognised that crystallographic disorder is not a reliable indicator of local dynamics in solution. Nevertheless, differences in the rate of ion transport across the 3-fold channels of the variant *versus* wild-type proteins may result from loss of potential gradient associated with the negatively charged headgroup of Asp rather than changes in local mobility. To investigate this further, electrostatic surface potentials were calculated for wild-type and D131N variants of both HuHF and FtMt (Fig. S4). These show that for both proteins there is a loss of negative potential upon substituting Asp131. Interestingly, the impact is greater for HuHF than for FtMt, consistent with the observed impact on metal binding in the channels and FOC.

The Fe^2+^/Mg^2+^-ferritin co-crystals used in this study contained metal ions bound at their equilibrium ratio prior to exposure to O_2_. This equilibrium was disrupted by the initiation of Fe^2+^/O_2_ reactivity on O_2_ exposure and changes in metal ion occupancy over time report on the extent to which fresh substrate can be transported through the protein to maintain the initially observed ion binding status. Therefore, the time dependence of the occupancies of the metal-binding sites reported here suggest that the metal-binding affinity of the three-fold channel of HuHF was disrupted by the D131N substitution to a greater extent than that of FtMt. Consequently, transport of substrate through the HuHF cage was also impaired to a greater extent meaning that iron occupancy of the FOC was depleted at the longer O_2_ reaction time. This hypothesis was investigated further using absorbance-monitored solution kinetics measurements.

### Replacement of the three-fold channel metal-binding residue Asp131 significantly affects the access of Fe^2+^ to the FOC sites of both HuHF and FtMt

H-chain-type ferritins typically exhibit two kinetically distinct phases in their reactions with Fe^2+^ and O_2_. The first is the rapid, FOC-mediated oxidation of Fe^2+^ following its addition to apo protein. The second is only observed at stoichiometric ratios of iron to protein greater than the 48 required to fill all the FOC sites in the 24-mer. Under these conditions, for further Fe^2+^ to be oxidised at the FOC, the incumbent Fe^3+^ ions already bound to the FOCs must be cleared towards the central cavity for mineral core formation. Exit of Fe^3+^ from the FOC is orders of magnitude slower than the binding and oxidation of Fe^2+^ to this site, meaning that the rate of further oxidation is much slower during this mineralisation phase.

**Fig. 4:**
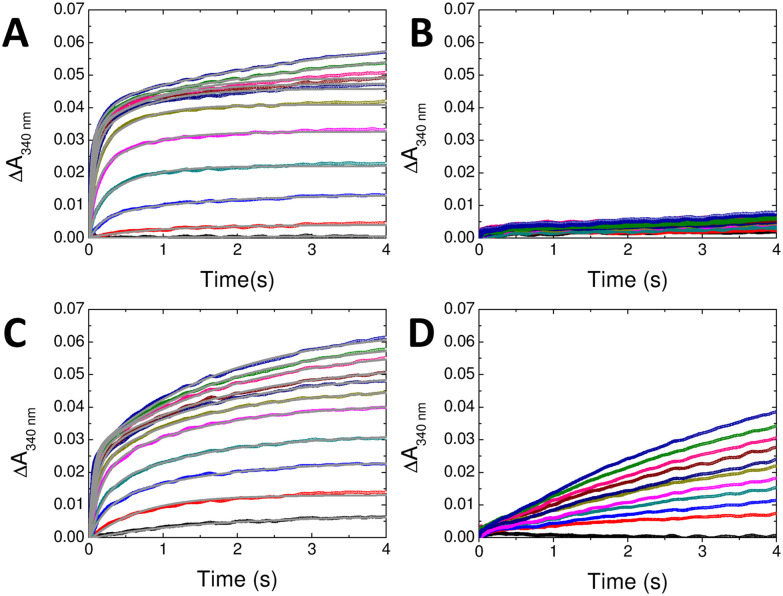
Rapid Fe^2+^ oxidation by HuHF, FtMt and respective D131N variants. Increase in 340 nm absorbance as a function of time following aerobic mixing of Fe^2+^ and wild-type HuHF (A), D131N HuHF (B), wild-type FtMt (C) and D131N FtMt (D). Final Fe^2+^ concentrations following mixing were 3 μM (black), 6 μM (red), 9 μM (blue), 12 μM (cyan), 15 μM (magenta), 18 μM (dark yellow), 21 μM (navy blue), 24 μM (dark red), 30 μM (pink), 36 μM (dark green) and 48 μM (royal blue). Final protein concentration was 0.5 μM throughout. Solid grey traces in panels (A) and (C) depict the (multi)exponential functions from which rate constants and amplitudes of Fe^2+^ oxidation phases were extracted.

O_2_-driven oxidation of Fe^2+^ at H-chain FOCs proceeds *via* a diferric-peroxo (DFP) intermediate that is hydrolysed to yield the diferric-oxo and H_2_O_2_ products of FOC activity. Both of these diferric species give rise to absorbance features centred at approximately 340 nm with an additional transient absorbance centred at approximately 650 nm due to DFP. FOC-catalysed oxidation of Fe^2+^ typically occurs on the timescale of several hundred milliseconds, meaning that the process can be conveniently studied by absorption spectroscopy in conjunction with stopped-flow mixing. Stopped-flow absorbance measurements at 340 nm of Fe^2+^ oxidation catalysed by wild-type HuHF resulted in the expected multiphasic reaction over several seconds following mixing of aerobic protein and Fe^2+^ ions (Fig. 4A). Fitting of the data using multi-exponential functions yielded apparent first-order rate constants for the rapid phase (Table S3). A plot of these as function of Fe^2+^ concentration yielded a second-order rate constant of 7.9 ± 0.4 × 10^5^ M^−1^ s^−1^ (Fig. S5), consistent with a first-order iron dependence for the rapid phase. Equivalent measurements with D131N HuHF demonstrated that very little Fe^2+^ oxidation occurred over the first 4 seconds of the reaction (Fig. 4B). The rapid phase was entirely lost and the rate and extent of the slower phase reduced such that extraction of rate constants from the data was not possible. The structural data presented above showed that the FOC of HuHF variant D131N remains competent to bind Fe^2+^ and therefore the significant impairment of Fe^2+^ oxidation demonstrates that Asp131 plays an important role in iron entry into HuHF and access to the FOC sites.

Equivalent stopped-flow measurements were performed with wild-type FtMt and its D131N variant. For wild-type FtMt, a mechanistic study of Fe^2+^ oxidation and mineralisation was recently reported, demonstrating significant mechanistic differences to HuHF,^22^ despite their very close sequence similarity. Nevertheless, oxidation was still characterised by rapid and slower phases (Fig. 4C). Fitting of the data using multi-exponential functions generated first-order rate constants for the rapid phase following increasing additions of Fe^2+^ to the apo protein (Fig. S5) yielding a second-order rate constant of 4.4 ± 0.1 × 10^5^ M^−1^ s^−1^. Again, the D131N substitution resulted in loss of the rapid phase of Fe^2+^ oxidation but, in contrast to HuHF, a significant proportion of the added Fe^2+^ was still oxidised, albeit at a slower rate, within the first 4 seconds of the reaction (Table S3). This slower phase of Fe^2+^ oxidation in D131N FtMt occurred at a rate that was independent of Fe^2+^ concentration, indicating that binding of Fe^2+^ was no longer rate limiting for FOC turnover in this variant. These data suggest that the D131N substitution impacts the function of the three-fold channel to the extent that the mechanism of iron oxidation is affected such that it becomes rate limiting. Consistent with this, neither protein gave rise to the transient 650 nm absorbance characteristic of DFP formation (Fig. S6), suggesting that the rate of formation of this intermediate is insufficient for it to accumulate during the catalysis of O_2_-driven Fe^2+^ oxidation by the D131N variants. Quantitation of the rate of Fe^2+^ translocation from bulk solution to the FOC is only possible for the wild type proteins, meaning that it is not possible to extract a full mechanistic model of iron transport from the data presented. Nevertheless, the relative importance of Asp131 in the two proteins was apparent.

**Fig. 5:**
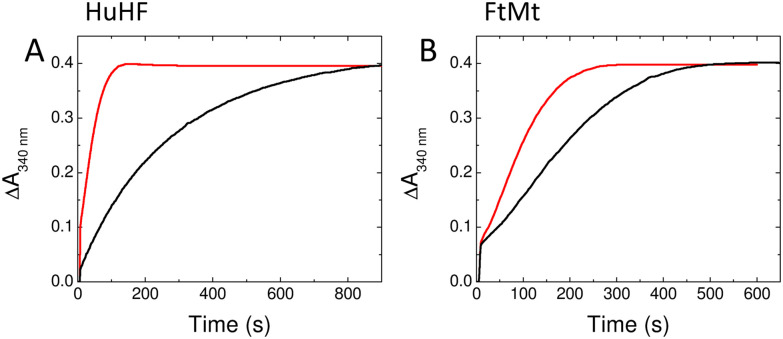
Iron mineralisation in HuHF, FtMt and respective D131N variants. The increase in 340 nm absorbance following aerobic mixing of HuHF (A) or FtMt (B) with 400 equivalents of Fe^2+^. Black traces represent data for the D131N variants, red traces data for the wild-type proteins for comparison.

Biomineralisation of iron within the core of ferritin can also be monitored by exploiting the increase in absorbance at 340 nm, with the process typically occurring on the timescale of several minutes. The absorbance at 340 nm of HuHF, FtMt and the D131N variants of each as a function of time following aerobic mixing of the proteins with 400 Fe^2+^ per cage is shown in Fig. 5. The impact of the D131N substitution on the rate of mineral core formation was significantly greater for HuHF than for FtMt (Table S4), suggesting that function of the three-fold channel is impaired to a greater degree by the D131N substitution in the former. This is consistent both with the structural data and predictions based on the relative rates of FOC-catalysed Fe^2+^ oxidation presented above.

### The D131N substitution impacts iron release from HuHF but not from FtMt

The three-fold channel is implicated in iron release from animal ferritins in addition to its entry into the proteins.^17^ Therefore, it was of interest to investigate whether rates of iron release were also affected by the D131N substitution as both activities may be required for the protein to function as a dynamic store of iron. Proteins were incubated with 16 successive aliquots of 50 Fe^2+^ per cage, with iron oxidation allowed to reach completion between additions, such that at the endpoint each protein had a theoretical maximum loading of 800 Fe^3+^ per cage. This iron loading protocol was designed to match the increments in iron loading to the number of FOC iron-binding sites, thereby minimising the potential for reaction between Fe^2+^ in solution and the peroxide generated by FOC activity during previous Fe^2+^ additions.

**Fig. 6:**
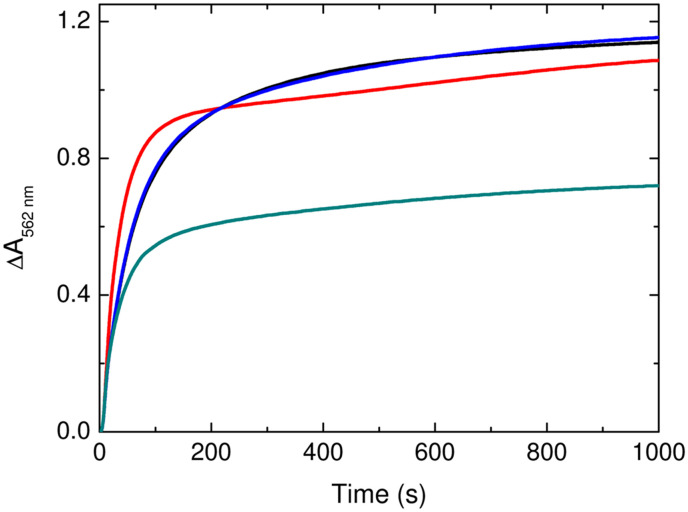
Iron release from HuHF, FtMt and respective D131N variants. Plots of the increase in 562 nm absorbance as a function of time following the anaerobic mixing of sodium dithionite with a solution of ferrozine and iron-loaded wild-type FtMt (black), wild-type HuHF (red), D131N FtMt (blue) or D131N HuHF (cyan).

Iron loading of each of the proteins was determined by colorimetric analysis following acid digestion of the ferritin cages (see Experimental section below). The intensity of the absorbance at 595 nm due to formation of the Fe^2+^ complex with 3-(2-pyridyl)-5,6-di(2-furyl)-1,2,4-triazine-5′,5″-disulfonic acid (ferene) was compared to that from equivalently treated standard Fe^2+^ solutions (Fig. S7). The data confirmed that, despite their differing rates of iron mineralisation, each protein formed a core of almost identical size with an average of approximately 550 Fe per cage, significantly below the theoretical maximum. The rate and extent of iron release from each of the proteins was determined from the increase in 563 nm absorbance due to the formation of the complex of Fe^2+^ with 3-(-2-pyridyl)-5,6-bis(4-phenylsulfonic acid)-1,2,4-triazine (ferrozine) following the addition of sodium dithionite to anaerobic solutions containing ferritin and ferrozine (Fig. 6). The data show that iron release from FtMt was unaffected by the D131N substitution, with approximately 90% of mineralised iron released from both the wild-type and variant protein with identical kinetics (Table S4). In contrast the initial rate and extent of iron release from HuHF decreased by approximately 50% in variant D131N compared to the wild-type protein.

## Conclusions

Ferritins located within the cytosol of animal cells are thought to act as buffers for the concentration of chelatable Fe^2+^. This requires the ability to rapidly sequester excess Fe^2+^ from solution, oxidise it to Fe^3+^ for safe storage in a chemically inert form, and to release reduced iron back to solution should the concentration of the labile iron pool become depleted.^8^ As the conduit for transport of Fe^2+^ across the protein coat, the three-fold channels are critical for all these processes. The peptide sequence of the three-fold channel in cytosolic ferritins from a variety of animals is highly conserved (Fig. S1), including the strictly conserved residues Asp131 and Glu134 (HuHF numbering). This, together with a number of reports detailing the consequences of the disruption of this twin carboxylate motif in the H′ subunit (also known as M-chain) of ferritin from *Rana catesbeiana*,^23–25^ led to the conclusion that both residues were required to enable rapid transport of Fe^2+^ across the three-fold channel. However, a subsequent report on the bacterial ferritin *Syn*Ftn (from the marine cyanobacterium *Synechococcus* CC9311) demonstrated rapid transport of Fe^2+^ from bulk solution to the FOC *via* three-fold channels that feature a carboxylate only at the equivalent position to Asp131 of HuHF, with the equivalent to Glu134 being Val.^26,27^ Thus, the observation of natural variation at position 131 in mitochondrial ferritin raised the possibility that the three-fold channel of animal ferritins may also function with a single carboxylate. However, in this instance the remaining carboxylate would be at position 134 with Asp131 replaced by Asn, rather than Ala or Glu as commonly used in site directed mutagenesis for activity studies.^21,24,25,27^

The data presented here demonstrate that transport of Fe^2+^ across the three-fold channel is severely inhibited by the D131N substitution in both HuHF and FtMt, although the impact is greater in the former. However, due to the vast difference in the intrinsic rates of iron binding, oxidation, mineralisation and release in animal ferritins, only the binding and oxidation of Fe^2+^ are compromised by the reduced activity of the three-fold channel in D131N FtMt. In contrast, activity of the channel in D131N HuHF is impaired sufficiently to impact all the above processes. We note that the relative impacts of the substitution are correlated with the greater impact on electrostatic potentials in the three-fold channels of HuHF.

Thus, rapid Fe^2+^ uptake *via* three-fold channels is more important for the activity of HuHF than for FtMt. This seems counter-intuitive since, whilst HuHF acts as a dynamic iron store, FtMt functions in oxidative stress response^28^ where the rapid sequestration of the potential Fenton reagent Fe^2+^ might be expected to be crucial. However, recent studies of human FtMt revealed unusual mechanistic features, with the FOC of the protein poised in a Fe^2+^/Fe^3+^ mixed valent state, a form that is highly reactive toward O_2_. This led to the suggestion that the role of mitochondrial ferritin is to act as a buffer of O_2_ during impaired respiratory function.^22^ Such activity would not be critically dependent on rapid transport of Fe^2+^ from bulk solution to the FOC. Together with the fact that the D131N variant of human FtMt retained at least some capacity to take up Fe^2+^ rapidly (Fig. 4), this may account for the existence, at least in the case of the protein from tarsier, of a naturally occurring variation (Asn in place of Asp131) in the FtMt three-fold channel. We would assume that uptake of Fe^2+^ by this protein would be affected similarly to the D131N variant of human FtMt (∼82% identity between the mature proteins) studied here, consistent with the conclusion that Asp131 is more important for the function of HuHF than it is for FtMt. Nonetheless, the data presented here support the conclusion that the twin carboxylate motif is required for rapid transport of Fe^2+^ across the 3-fold channel. The fact that a natural substitution of one of the carboxylates occurs in the tarsier FtMt indicates that the rapid transport of Fe^2+^ across the 3-fold channel is less critical to the function of FtMts than it is to their cytosolic equivalents.

## Experimental

### Protein expression and purification

Plasmids encoding HuHF, FtMt and respective D131N variant proteins in the pET21a expression vector were purchased from Genscript (Netherlands). Both FtMt constructs lacked the predicted mitochondrial targeting sequence and first 9 amino acid residues at the N terminus of the mature protein. Proteins were expressed from *Escherichia coli* strain BL21 λDE3. Cells were spread onto Lysogeny Broth (LB) agar plates containing 100 µg mL^−1^ ampicillin and incubated overnight at 37 °C prior to picking single colonies into liquid media. All cultures in liquid media contained 100 µg mL^−1^ ampicillin and were grown at 37 °C, 200 rpm shaking unless otherwise stated. Single colonies were picked into 5 mL LB and grown on for 8 h. 400 µL of the resulting cell culture was added to 80 mL of LB which was incubated overnight. 50 mL of the overnight culture was added to 5 L of LB and grown on until OD_600_ = 0.6–0.8, at which point protein expression was induced by addition of isopropyl β-d-1-thiogalactopyranoside (IPTG) to final concentration of 1 mM. Cultures were grown overnight at 30 °C, 90 rpm shaking before harvesting cells by centrifugation.

Cells were resuspended in 20 mM HEPES, 100 mM KCl, 0.1 mM EDTA, pH 7.8 (buffer A) then lysed by sonication. Insoluble material was removed by centrifugation at 40 000*g*, 4 °C for 45 min. Thermally unstable proteins were removed from supernatant by heating to 65 °C for 15 min followed by a further round of centrifugation. Ferritin was then precipitated from the soluble fraction by addition of ammonium sulfate to a final concentration of 0.55 g mL^−1^ and collected *via* a final round of centrifugation. The precipitated ferritin was re-dissolved in the minimum volume of buffer A before dialysing against 1 L of identical buffer overnight. Contaminating proteins were removed using size-exclusion chromatography (HiPrep 26/60 Sephacryl S-300HR, Cytiva) and contaminating DNA removed using anion exchange chromatography (HiTrap Q FF, Cytiva) with samples loaded in buffer A before eluting by stepping to 30% buffer B (20 mM HEPES, 100 mM KCl, 0.1 mM EDTA, 1 M NaCl, pH 7.8). Residual solid material was removed prior to chromatography steps by filtering through a 0.22 μm membrane (StarLab UK).

Proteins as isolated contained small amounts of iron that were removed using the method of Bauminger *et al.*^29^ Iron-free protein was exchanged into 100 mM MES pH 6.5 by centrifugation over a 10 kDa molecular weight cut off cellulose membrane (Millipore). The purity of the resulting protein was confirmed by SDS-PAGE and judged free from DNA contamination once the absorbance at 280 nm was ≥1.5 times that at 260 nm. Protein concentration was determined by absorbance using *ε*_280_ = 408 000 M^−1^ cm^−1^.^11^

### Protein crystallization and structure solution

Protein crystallisation was performed in a nitrogen-filled chamber (Belle technology, [O_2_] < 10 ppm). Wild-type FtMt (2 mg mL^−1^), HuHF (10 mg mL^−1^), FtMt D131N (2.4 mg mL^−1^) and HuHF D131N (12 mg mL^−1^) were exchanged into 20 mM MES pH 6.5 and 2 µL drops mixed with equal volume of well solution: 100 mM bicine, 100 mM NaCl, 2 M MgCl_2_, 60 mM FeCl_2_, 3 mM NaN_3_, 150 mM NaCl, pH 9.0 for FtMt; or, 100 mM bicine, 100 mM NaCl, 2 M MgCl_2_, 60 mM FeCl_2_, 150 mM NaCl, pH 9.0 for HuHF. Protein solutions in sitting drops were equilibrated with 200 µL of the well solution by vapour diffusion at 16 °C. Crystals with bi-pyramidal symmetry appeared within 24 h and grew to 100–200 µm within two weeks. Crystals were transferred to cryoprotectant comprised of well solution with 30% glycerol and MgCl_2_ concentration increased to 2.2 M for short (2–5 min) or long (30–40 min) O_2_ exposure before flash freezing in liquid nitrogen.

Data collection was performed at the Diamond Light Source on beamline i24 using X-rays of wavelength 0.975 Å for high resolution datasets (high energy dataset), 1.74 Å for the iron K-edge absorption maximum (peak dataset), and 1.75 Å for the low energy side of the iron K-edge absorption maximum (low energy dataset). All data were indexed and processed using XDS and Aimless as part of the automatic xia2 pipeline.^30^ Statistics are summarised in Table S1 for high energy data and in Table S2 for data used for calculation of Bijvoet-difference Fourier (anomalous-scattering-density) maps.

Structure solution was performed by molecular replacement using phenix.phaser MR.^31^ The 1.7 Å resolution structure of wild-type FtMt,^32^ PDB entry 1R03, was used as the search model for wild-type and D131N FtMt, and the 1.7 Å resolution structure of wild-type HuHF,^33^ PDB entry 5N27, as the search model for the corresponding HuHF proteins. In all cases the asymmetric unit contained a single copy of the protein monomer. Placement of metal ions was performed by reference to Bijvoet-difference Fourier maps calculated from anomalous scattering data measured at the iron K-edge absorption peak wavelength (Table S2). Metal placement was confirmed by reference to the corresponding Bijvoet-difference Fourier maps calculated using the low energy datasets. Model refinement employed iterative cycles using phenix.refine and manual correction using WinCOOT^34^ employing data collected at the high energy wavelength. No metal coordination restraints were applied to metal sites during refinement. Anisotropic temperature factor refinement was employed for all metal ions and their occupancies were manually adjusted to ensure that the average *B* factor of the metal fell within ±15% of the *B* factors of atoms in their immediate environment. Occupancies relating to the metal binding sites in the refined structures can be found in Table 1.

### Electrostatic calculations

PQR files were prepared from the corresponding PDB file of proteins exposed to O_2_ for 5 min, with metal and chloride ions removed, using PDB2PQR with the CHARMM forcefield and protonation states calculated at pH 6.5. Electrostatic surface maps were generated from the PQR files by solution of the Poisson–Boltzmann equation using APBS^35^ with default parameters. The resulting electrostatic potential maps were visualized in ChimeraX.^36^

### Absorbance-monitored kinetic studies

Ferritin-catalysed iron oxidation was measured by change in absorbance at 340 nm due to a ligand to metal charge transfer transition following O_2_-driven oxidation of Fe^2+^ to Fe^3+^. Iron mineralisation activity of the wild-type and variant proteins was determined by the rate of increase of absorbance measured using a Hitachi U-2900 spectrophotometer with the sample chamber equilibrated at 25 °C. Ferrous ammonium sulfate in 1 mM HCl was added to 0.5 µM protein in 100 mM MES pH 6.5 to a final concentration of 200 µM in a 1 cm pathlength cuvette. Initial rate of iron mineralisation was calculated from the gradient of the linear portion of plots of 340 nm absorbance as a function of time. The rate of iron oxidation at FOCs was determined from the rate of the increase in absorbance at 340 nm following aerobic mixing of Fe^2+^ and protein using an Applied Photophysics Bio-sequential DX.17MV spectrophotometer with sample chamber equilibrated at 25 °C. 1 µM protein in 100 mM MES pH 6.5 was mixed with an equal volume of ferrous ammonium sulfate at concentrations of 6, 12, 18, 24, 30, 36, 42, 48, 60, 72 and 96 µM in 1 mM HCl. OriginPro 2024 (OriginLab) was used to fit traces of absorbance as a function of time to bi-exponential decay functions representing rapid (r) and slow (s) phases of Fe^2+^ oxidation.

1 Δ*A*_340_(*t*) = Δ*A*^(tot)^_340_ − Δ*A*^r^_340_*e*^−*k*_r_*t*^ − Δ*A*^s^_340_*e*^−*k*_s_*t*^

Turnover of FOCs was also monitored by recording absorbance at 650 and 340 nm following the aerobic mixing of 8 µM protein in 100 mM MES pH 6.5 with 384 µM ferrous ammonium sulfate in 1 mM HCl using an Applied Photophysics Bio-sequential DX.17MV with sample chamber equilibrated at 25 °C. Increase in 340 nm absorbance as a function of time reports on oxidation of Fe^2+^ to Fe^3+^, whilst the time dependence of the 650 nm absorbance is a specific probe of the formation and decay of the DFP intermediate formed during FOC activity.

The rate of release of Fe^2+^ following the reduction of ferritin encapsulated ferric mineral cores was determined *via* the formation of a coloured complex (*ε*_562 nm_ = 27 000 M^−1^ cm^−1^) with ferrozine. Samples were prepared by sequential additions of 50 equivalents of Fe^2+^ to 0.5 μM solutions of ferritin in 100 mM MES pH 6.5. Proteins were loaded with 16 aliquots in total such that the nominal iron loading of each sample was 800 equivalents of Fe^3+^ per cage. Precipitated material was removed by centrifugation at 17 000*g* for 10 min following the 8^th^ and 16^th^ additions and the soluble fraction stored at −80 °C until use.

Because variant and wild-type proteins exhibited significant differences in Fe^2+^ oxidation and iron mineralisation activities, aliquots of each sample were subjected to acid digestion and iron content analysis to ensure that comparable mineral core sizes were formed in each case. 0.25 mL of iron-loaded protein was incubated at 37 °C overnight with an equal volume of 68% HNO_3_. Samples were neutralised by addition of 2 mL of a saturated ammonium acetate solution prior to addition of 0.4 mL of 5% w/v sodium ascorbate and 0.4 mL 10 mM ferene. Following incubation for 30 min to allow formation of the iron–ferene complex, iron concentration was calculated from the absorbance at 595 nm of the Fe^2+^/ferene complex using the relationship [Fe] (μM) = (*A*_595_ − 0.0397)/0.0052 determined by equivalent treatment of iron solutions of known concentration (Fig. S7). This analysis indicated actual iron loadings of 578 (HuHF), 539 (FtMt), 557 (D131N HuHF) and 510 (D131N FtMt) per ferritin cage.

Samples of each protein were diluted into an anaerobic mixed buffer system (10 mM potassium acetate, 10 mM MES, 10 mM MOPS, 10 mM Tris, 200 mM NaCl, pH 6.0) containing 1 mM ferrozine such that the final mineralised iron concentration was approximately 45 μM based on the iron loadings calculated above. 400 μL aliquots were sealed into anaerobic cuvettes and the rate and extent of iron release from the ferritin determined by the increase in absorbance at 563 nm following injection of sodium dithionite to a final concentration of 100 μM.

## Author contributions

ZB: investigation, formal analysis, supervision, writing – original draft, CH: investigation, formal analysis, AMH: investigation, formal analysis, supervision, writing – review and editing, JMB: conceptualisation, investigation, formal analysis, supervision, writing – review and editing, NLB: funding acquisition, supervision, writing – review and editing.

## Conflicts of interest

There are no conflicts to declare.

## Supplementary Material

DT-055-D5DT02739J-s001

## Data Availability

Coordinate files detailing the structures derived from X-ray diffraction data can be downloaded from the protein data bank (https://www.rcsb.org) using the accession codes listed in Table S1 of the supplementary information (SI). Supplementary information is available. See DOI: https://doi.org/10.1039/d5dt02739j. Other data for this article, including stopped-flow and standard kinetic data, and absorbance data, are available at OSF at https://doi.org/10.17605/OSF.IO/7GC8M.
