# Supplementary material for: Impact of the three-fold channel substitution D131N on kinetics of translocation of Fe2+ across the protein coat is more severe for human cytosolic H-chain ferritin than for human mitochondrial ferritin
Source: Dalton Trans. 2026 Apr 1;55(17):6844–53. doi: 10.1039/d5dt02739j (PMC13088775; doi:10.1039/d5dt02739j)
Supplement: DT-055-D5DT02739J-s001 [file DT-055-D5DT02739J-s001.pdf]

Supplementary Information (SI) for Dalton Transactions.
This journal is © The Royal Society of Chemistry 2026

Supplementary Information

Impact of the three-fold channel substitution D131N on kinetics of translocation of Fe^{2+} across the protein coat is more severe for human cytosolic H-chain ferritin than for human mitochondrial ferritin.

Zinnia Bugg, Charlie Hazlewood, Andrew M. Hemmings, Justin M. Bradley and Nick E. Le Brun

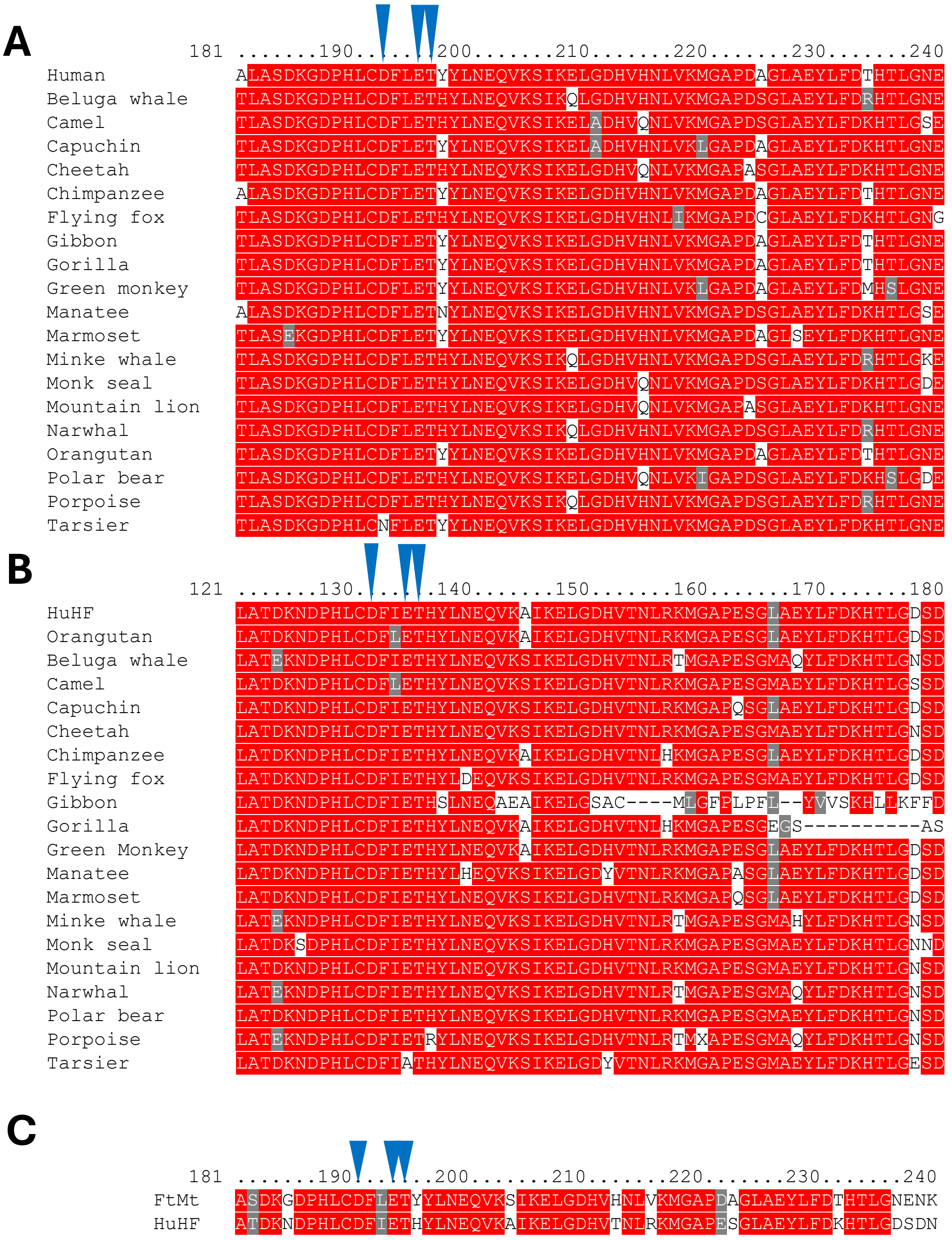

Figure S1. Sequence alignments of cytosolic and mitochondrial ferritins. Alignments of the peptide sequences of predicted mitochondrial (**A**) or cytosolic H-chain (**B**) ferritins from the organisms listed showing the region around the three-fold channel. A similar alignment between the mitochondrial & cytosolic H-chain ferritns of humans is shown in (**C**). Blue triangles mark the positions of residues observed to coordinate metal ions in structures derived from X-ray diffraction data.

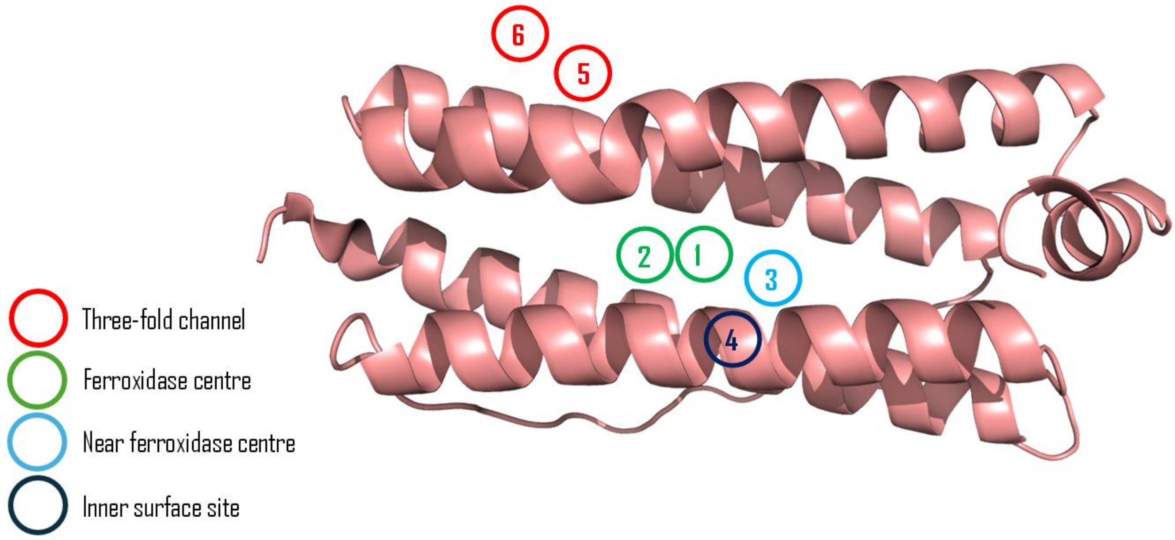

Figure S2. Locations of metal binding sites 1-6 within the ferritin subunit.

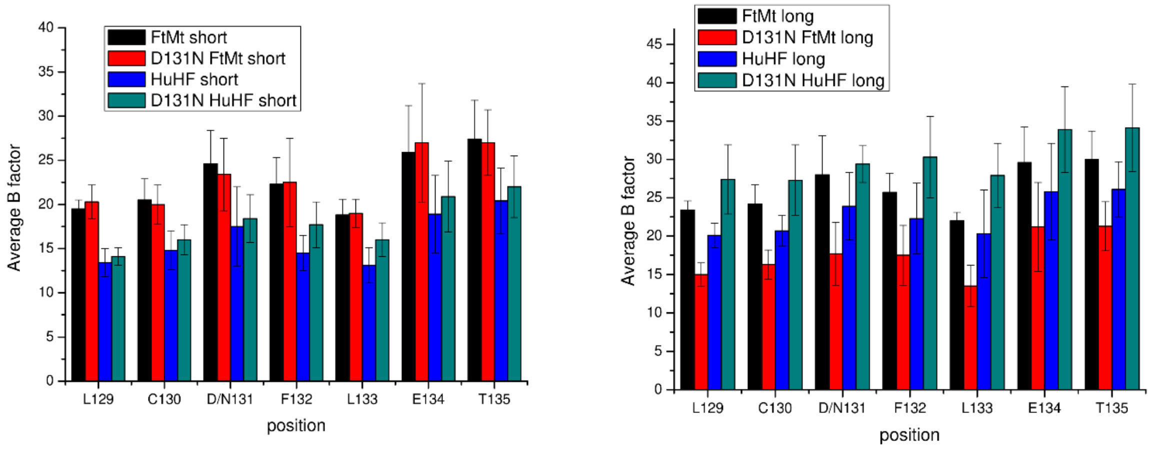

Figure S3. **Analyses of average B factors associated with three-fold channel residues in structures of HuHF, FtMt and D131N variants.**

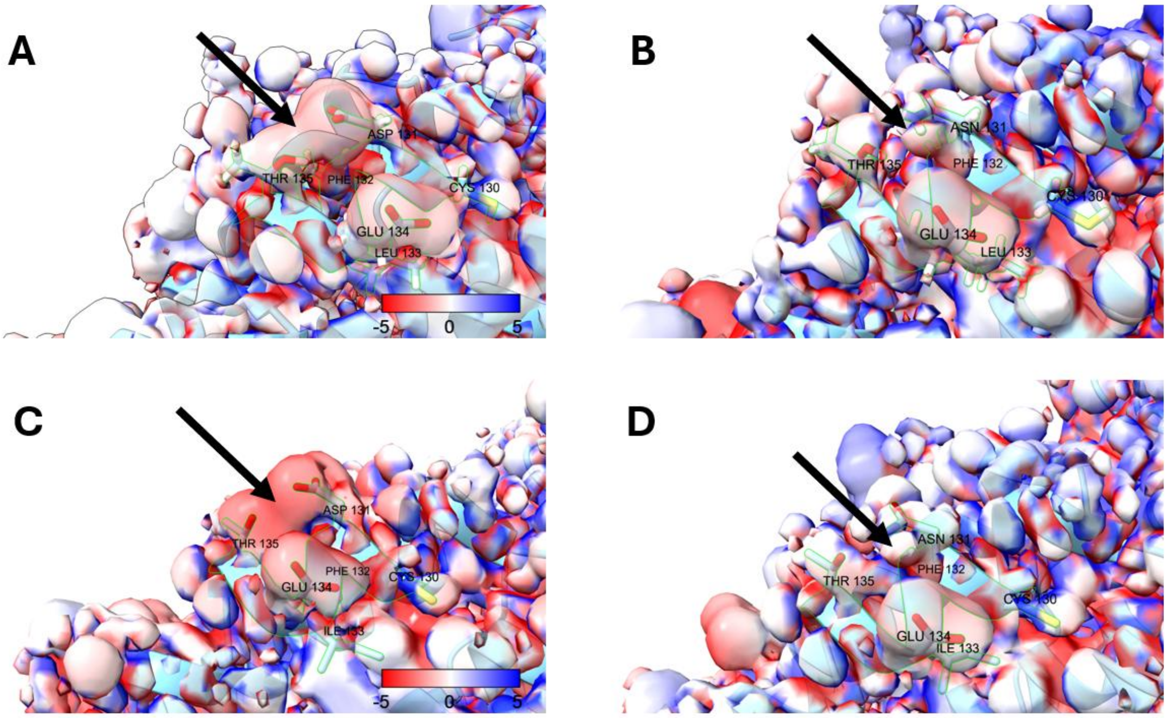

Figure S4. Electrostatic potential surface calculations. Electrostatic potential surfaces calculated for iron loaded FtMt (**A**), D131N FtMt (**B**), HuHF (**C**) and D131N HuHF (**D**) exposed to O_2 for 5 min. All surfaces are rendered between the limits -5 to +5 RTe_c^{-1} and maps coloured according to the key displayed in panels (A) and (C). Of note is that the negative potential in the region between residues 131 and 135 (highlighted by the black arrows) is greater for wild-type HuHF than for FtMt. This may arise from a slightly different conformation of Thr135 in wild-type HuHF compared to wild-type FtMt, resulting in greater negative electrostatic potential at the inner exit of the three-fold channel in the former. The electrostatic potential is abolished by D131N substitution in both proteins, but this is presumably of greater consequence in HuHF than in FtMt due to the magnitude of the negative potential that is lost being greater.

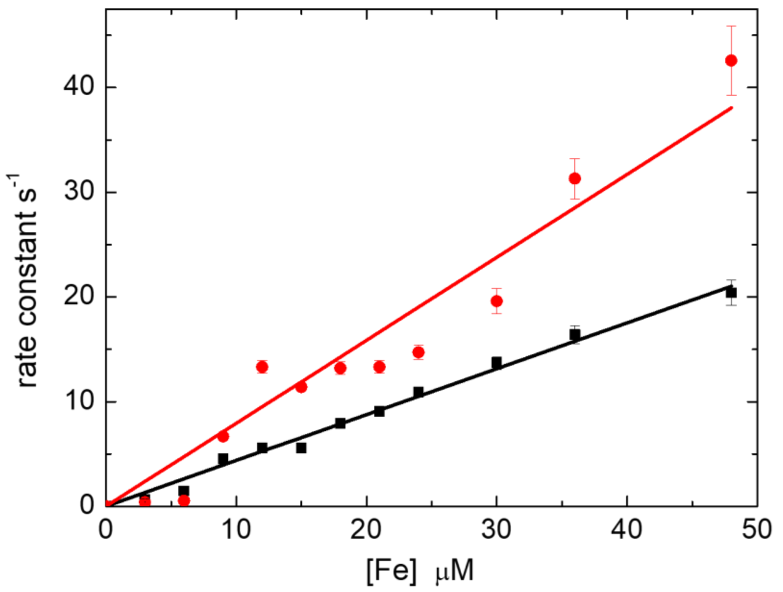

Figure S5. Dependence of rapid Fe^{2+} oxidation rate constant on Fe^{2+} concentration. Linear dependence of the rate constant describing rapid Fe^{2+} oxidation catalysed by FtMt (black squares) and HuHF (red circles). Error bars are 3× the standard error estimated from least squares fitting of exponential functions through the data shown in Figure 4. Solid lines represent the best fit to a straight line through the origin.

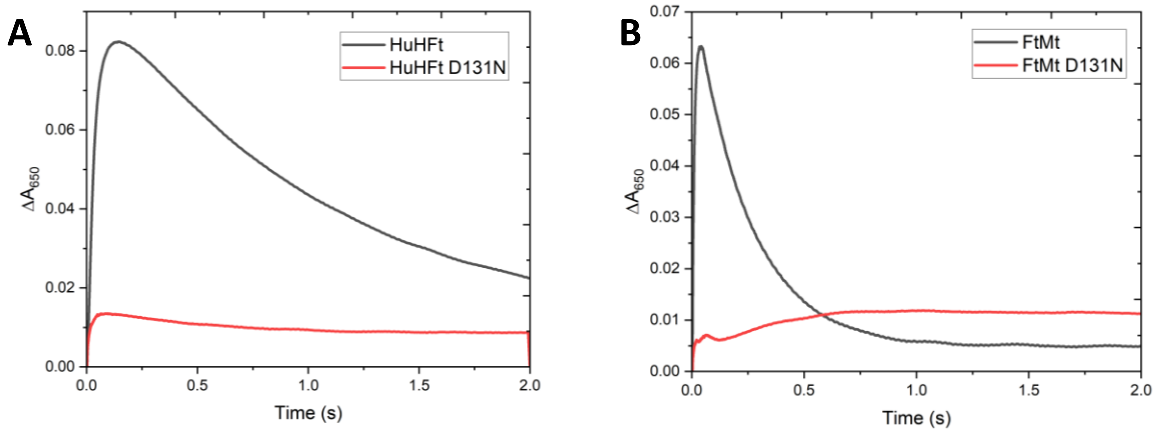

Figure S6. DFP formation in HuHF, FtMt and D131N variants. Absorbance at 650 nm as a function of time following the aerobic mixing of (**A**) wild-type or variant D131N HuHF, or (**B**) wild type or variant D131N FtMt with 48 Fe^{2+} per cage. Transient absorbance at this wavelength in the wild-type but not the variant proteins indicates that the DFP intermediate accumulates in the former but not the latter.

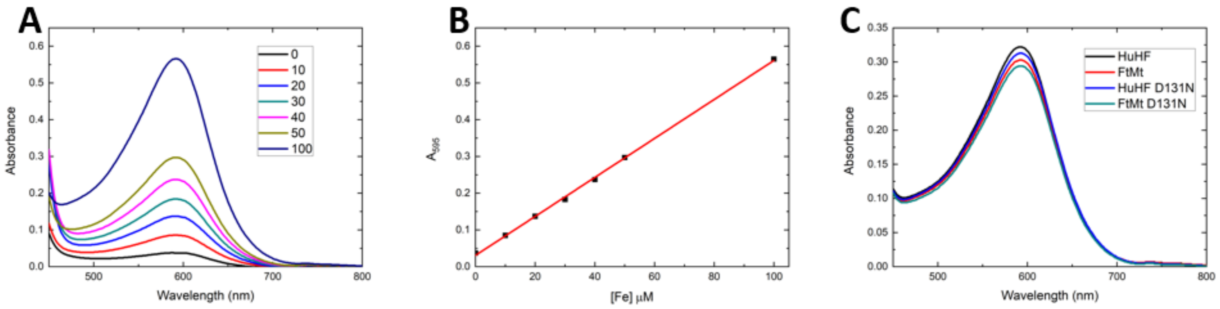

Figure S7. Iron content of iron-loaded samples of HuHF, FtMt and respective D131N variants used in iron release assays. (**A**) absorbance spectra of standard Fe^{2+} solutions treated equivalently to acid digested ferritin samples. (**B**) The linear relationship between 595 nm absorbance and Fe^{2+} concentration for the samples from (A). (**C**) Absorbance spectra of the complex between ferene and Fe^{2+} released during acid digestion of HuHF (black line), FtMt (red), HuHF D131N (blue) and FtMt D131N (cyan).

Table S1. High resolution data collection and refinement statistics.

Sample name	HuHF 5 min O_2 soak	HuHF 20 min O_2 soak	D131N HuHF 5 min O_2 soak	D131N HuHF 20 min O_2 soak	FtMt 5 min O_2 soak	FtMt 20 min O_2 soak	D131N FtMt 5 min O_2 soak	D131N FtMt 20 min O_2 soak
PDB entry code	9I1C	9I1B	9I19	9I1E	9HYC	9I1A	9I1D	9I1F
Beamline	DLS i24	DLS i24	DLS i24	DLS i24	DLS i24	DLS i24	DLS i24	DLS i24
Wavelength/ Å	0.975	0.975	0.975	0.975	0.975	0.975	0.975	0.975
Resolution range	41.98-1.582 (1.638-1.582)	54.93-1.63 (1.689-1.63)	42-1.631 (1.689-1.631)	55.37-1.76 (1.823-1.76)	55.15-1.711 (1.772-1.711)	55-1.73 (1.792-1.73)	40.88-1.651 (1.71-1.651)	27.55-1.72 (1.782-1.72)
Space group	F 4 3 2	F 4 3 2	F 4 3 2	F 4 3 2	F 4 3 2	F 4 3 2	F 4 3 2	F 4 3 2
Unit cell	182.98	182.18	183.09	183.66	182.90	182.41	182.83	182.71
Total reflections	3041242 (5800)	3012019 (22137)	3051139 (22927)	2991726 (81677)	3006115 (61214)	2965400 (69394)	3020629 (31099)	3004611 (62043)
Unique reflections	34509 (1931)	32168 (2694)	32092 (2671)	26854 (2582)	28771 (2744)	27662 (2670)	31209 (2646)	28132 (2595)
Multiplicity	88.1 (3.0)	93.6 (8.2)	95.1 (8.6)	111.4 (31.6)	104.5 (22.3)	107.2 (26)	96.8 (11.8)	106.8 (23.9)
Completeness (%)	94.82 (53.04)	97.97 (84.40)	96.24 (81.13)	99.55 (95.54)	99.35 (93.67)	99.41 (94.28)	97.14 (80.73)	99.32 (94.09)
Mean I/sigma(I)	39.24 (0.69)	36.09 (0.87)	29.19 (0.78)	15.78 (0.51)	22.32 (1.08)	17.83 (0.62)	32.57 (0.84)	51.21 (3.96)
Wilson B-factor	16.73	20.32	18.24	28.75	22.52	24.46	21.27	17
R-merge	0.1068 (0.9225)	0.1187 (1.436)	0.146 (1.624)	0.3159 (6.765)	0.2414 (4.602)	0.2461 (3.608)	0.1324 (1.882)	0.1027 (0.6636)
R-meas	0.1074 (1.141)	0.1193 (1.534)	0.1467 (1.725)	0.3173 (6.876)	0.2425 (4.704)	0.2472 (3.68)	0.133 (1.971)	0.1031 (0.6779)
R-pim	0.009841 (0.6435)	0.01079 (0.512)	0.0134 (0.5569)	0.02865 (1.211)	0.02218 (0.9268)	0.02226 (0.7017)	0.01204 (0.5565)	0.009079 (0.1312)
CC1/2	1 (0.357)	1 (0.423)	0.999 (0.452)	1 (0.399)	1 (0.497)	0.999 (0.396)	1 (0.456)	1 (0.909)
CC*	1 (0.725)	1 (0.771)	1 (0.789)	1 (0.755)	1 (0.815)	1 (0.753)	1 (0.791)	1 (0.976)
Reflections used in refinement	34464 (1891)	32155 (2684)	32052 (2635)	26755 (2486)	28671 (2649)	27560 (2569)	31098 (2535)	28130 (2595)
Reflections used for R-free	1755 (99)	1675 (129)	1625 (134)	1385 (132)	1429 (137)	1353 (119)	1581 (121)	1991 (183)
R-work	0.1557 (0.3344)	0.1857 (0.3304)	0.1663 (0.3505)	0.1585 (0.3489)	0.1668 (0.2946)	0.1826 (0.3462)	0.1608 (0.3237)	0.1647 (0.2119)
R-free	0.1860 (0.3415)	0.2139 (0.3493)	0.1941 (0.3928)	0.1904 (0.3649)	0.1945 (0.3113)	0.2168 (0.3442)	0.1842 (0.3461)	0.1886 (0.2627)
CC(work)	0.967 (0.726)	0.944 (0.727)	0.965 (0.792)	0.964 (0.734)	0.956 (0.847)	0.949 (0.749)	0.955 (0.792)	0.957 (0.914)
CC(free)	0.951 (0.49)	0.914 (0.672)	0.953 (0.776)	0.948 (0.687)	0.948 (0.864)	0.937 (0.629)	0.952 (0.781)	0.944 (0.873)
Number of non-hydrogen atoms	1766	1560	1709	1705	1619	1556	1607	1689
macromolecules	1436	1334	1424	1489	1383	1363	1392	1416
ligands	17	10	11	10	11	14	12	9

solvent	313	216	274	206	225	179	203	264
Number of protein residues	173	163	174	174	168	167	168	168
RMS(bonds)	0.006	0.011	0.006	0.01	0.007	0.007	0.01	0.006
RMS(angles)	0.88	1.09	1.1	1.1	0.98	0.94	1.13	0.96
Ramachandran favored (%)	98.25	98.14	98.26	99.42	97.59	98.18	98.19	98.19
Ramachandran allowed (%)	1.75	1.86	1.74	0.58	2.41	1.82	1.81	1.81
Rotamer outliers (%)	0	0	0	0	0	0	0	0
Clashscore	1.41	14.83	3.21	2.36	3.69	2.24	3.29	5.38
Average B-factor (Å^2)	19.36	23.85	20.2	33.34	25.42	27.68	25.88	20.92
macromolecules	16.88	22.16	18.25	31.84	23.76	26.36	24.2	18.69
ligands	20.49	27.6	22.28	34.56	28.36	34.08	30.7	22.1
solvent	30.7	34.17	30.26	44.13	35.47	37.23	37.09	32.85

Table S2. Fe K_α absorption edge data collection and refinement statistics.

	**HuHF 2 min O_2 soak**	**HuHF 20 min O_2 soak**	**HuHF D131N 2 min O_2**	**HuHF D131N 20 min O_2**	**FtMt 2 min O_2**	**FtMt 20 min O_2**	**FtMt D131N 2 min O_2**	**FtMt D131N 20 min O_2**
Beamline	i24	i24	i24	i24	i24	i24	i24	i24
Wavelength/ Å	1.74	1.74	1.74	1.74	1.74	1.74	1.74	1.74
Resolution range	91.49 - 1.86 (1.89 - 1.86)	91.36 - 1.99 (2.02 - 1.99)	105.31 - 1.81 (1.84 - 1.81)	105.53 - 2.00 (2.03 - 2.00)	28.89 - 2.00 (2.04 - 2.00)	91.33 - 1.98 (2.01 - 1.98)	105.89 - 1.98 (2.01 - 1.98)	91.61 - 1.87 (1.90 - 1.87)
Total reflections	1233576	2393826	1197855	1201743	1114978 (23190)	1207548	1158410	1177622
Unique reflections	23937	18514	23434	68499	18143 (890	18770	19007	22174
Multiplicity	55.58 (6.87)	129.30 (52.81)	50.95 (2.53)	17.54 (7.94)	61.5 (26.1)	64.33 (24.67)	60.95 (24.60)	53.11 (8.56)
Completeness (%)	99.97 (99.46)	100.00 (100.000)	96.50 (53.60)	99.41 (93.38)	99.97 (99.44)	100 (100)	100 (100)	99.27 (93.19)
Anomalous multiplicity	30.26 (3.73)	70.69	27.8	9.1	33.2	35.17	33.3	28.89 (4.56)
Anomalous completeness (%)	99.65 (93.63)	100	95.35	98.85	100	100	100	99.16
<I>	6	11.8	4	2	3	9.8	1	3
<I/sI>	34.27	14.67	32.85	18.67	37.5	18.27	23.92	27.31
Rmerge	0.104	0.2937	0.101	0.4828	0.131	0.22	0.1709	0.1239
Rmeas	0.104	0.295	0.102	0.322	0.132	0.222	0.175	0.126
Rpim	0.018	0.025	0.013	0.054	0.016	0.027	0.022	0.022
CC(1/2)	0.9992	0.9992	0.9996	0.9958	1	0.999	0.9995	0.9995
CC(anom)	0.364	0.64	0.366	0.206	0.313	0.103	0.219	0.315
Anomalous signal resolution limit/ Å	2.45	2.41	2.34	2.56	2.53	3.64	3.32	2.57

Table S3. Iron oxidation at ferritin ferroxidase centres. Rate constants for Fe^{2+} oxidation extracted from exponential fitting of the time dependence of ΔA340 nm following aerobic mixing of ferritin at final concentration 0.5 µM with Fe^{2+} at the concentrations listed. Values in parentheses indicate the estimated uncertainty in the final significant figure. Oxidation in the first 4 s following mixing was not observed for D131N HuHF.

[Fe] (µM)	Wild-type FtMt		D131N FtMt		Wild-type HuHF	
	k_r (s^{-1})	k_s (s^{-1})	k_r (s^{-1})	k_s (s^{-1})	k_r (s^{-1})	k_s (s^{-1})
3	0.63(1)	-	-	-	0.4(1)	-
6	1.45(1)	-	-	0.29(3)	0.53(3)	-
9	4.5(1)	0.70(3)	-	0.10(3)	6.7(1)	0.80(2)
12	5.6(1)	0.70(2)	-	0.11(1)	13.3(2)	2.00(2)
15	5.6(1)	0.70(2)	-	0.11(1)	11.4(1)	1.70(2)
18	7.9(1)	0.70(2)	-	0.15(1)	13.2(2)	2.00(2)
21	9.1(1)	0.60(2)	-	0.13(1)	13.3(2)	1.5(1)
24	10.9(1)	0.50(1)	-	0.14(1)	14.7(3)	1.40(2)
30	13.7(2)	0.50(1)	-	0.14(1)	19.6(4)	1.10(3)
36	16.4(3)	0.60(1)	-	0.18(1)	31.3(6)	1.20(1)
48	20.4(4)	0.60(1)	-	0.20(1)	42.6(1)	1.10(1)

Table S4. Rates of iron mineralisation deduced from the time dependence of ΔA340 nm following aerobic mixing of 0.5 µM ferritin with 400 equivalents of Fe^{2+} or iron release deduced from the time dependence of ΔA563 nm following anaerobic addition of 100 µM sodium dithionite to ferritin containing approximately 600 Fe^{3+} per cage in the presence of ferrozine. Values in parentheses represent the estimated uncertainty in the final significant figure.

	Wild-type FtMt	D131N FtMt	Wild-type HuHF	D131N HuHF
Mineralisation rate (µM min^{-1})	60.0(6)	33.0(1)	135.0(3)	30.0(2)
Release rate (µM min^{-1})	23.6(7)	23.6(7)	32(3)	17(1)
